# Flow through the Emergency Department for Patients Presenting with Substance Use Disorder in Alberta, Canada

**DOI:** 10.5811/westjem.60350

**Published:** 2023-07-07

**Authors:** Jonah Edmundson, Kevin Skoblenick, Rhonda J. Rosychuk

**Affiliations:** * The King’s University, Department of Biology, Edmonton, Canada; † University of Alberta, Department of Emergency Medicine, Edmonton, Canada; ‡ Royal Alexandra Hospital, Edmonton, Canada; § University of Alberta, Neuroscience and Mental Health Institute, Edmonton, Canada; ¶ Women & Children’s Health Research Institute, Edmonton, Canada; || University of Alberta, Department of Pediatrics, Edmonton, Canada

## Abstract

**Introduction:**

Since 2016 the province of Alberta, Canada, has seen a significant increase in substance use disorder (SUD) presentations to the emergency department (ED) with a large surge during the COVID-19 pandemic. In this retrospective study we deconstruct the total length of stay (LOS) in the ED into stages for patients presenting with SUD and estimate the effects of covariates on the time to transition between stages.

**Methods:**

Using the Canadian Coding Standards for International Classification of Diseases, 10^th^ Modification, codes F10.0-F19.9 and T36.0-T50.9, we extracted data from the National Ambulatory Care Reporting System between April 1, 2019–March 31, 2020 on all ED presentations for SUD by Alberta residents. We used a multistate model to deconstruct the EDLOS into eight mutually exclusive states and determine which factors affected the time spent in each state.

**Results:**

We analyzed 66,880 presentations (37,530 patients). The mean age was 37.2 years, and 61% were male. The median total LOS in the ED was 6 hours 13 minutes. Patients presenting with methamphetamines (METH) intoxication and patients from low-income neighborhoods had significantly increased transition times between all states. Opposite this, opiate use was associated with faster transition times between almost all states. Metro EDs experienced slower transitions when attempting to discharge or admit patients when compared to urban or rural EDs. Emergency department crowding also had a dramatic effect on physician initial assessment times, while discharge and admission times in patients presenting with SUD were also significantly affected.

**Conclusion:**

Patients with SUD experience a variety of delays during their ED stay. Those with METH intoxication and those from the lowest income neighborhoods were most likely to experience slower transitions from state to state in the ED and may benefit from a focused approach to improve ED flow.

## INTRODUCTION

Emergency departments (ED) are seeing a steady increase in presentations related to substance use disorder (SUD).^1^ Since 2016 the province of Alberta, Canada, has seen a significant increase of SUD presentations to the ED with a large surge during the COVID-19 pandemic.^2^ These presentations are associated with increased resource utilization when compared to non-SUD presentations and increased healthcare expenditure costs, particularly in stimulant use disorder, such as methamphetamine (METH) use.^3^ Methamphetamine use and subsequent ED-related presentations are rapidly increasing in the US^4^–^6^ and Canada,^7^–^9^ accompanied by an increased ED length of stay (LOS) when compared to non-METH-related diagnoses.^10^ Similarly, alcohol use has seen a significant increase in ED presentations^11^ creating further issues due to its well-established prolonging effect on a patient’s ED LOS.^12^ It has been previously established that ED crowding contributes to poorer patient outcomes in all measured domains.^13^ With this increasing ED burden due to SUD, more research is required to improve ED flow for the SUD patient. By identifying bottlenecks in their transition to admission or discharge, optimal targets may be identified that would produce the most impactful change on their overall ED LOS and ED crowding.

The primary objectives of this retrospective study were to deconstruct the total LOS in the ED into stages (also known as states) through the use of a multistate model and to estimate the effects of covariates on the time to transition between states. A secondary objective was to determine how ED crowding may be associated with the time spent in each state.

## METHODS

### Study Design

In this population-based, retrospective cohort study we used data extracted from the National Ambulatory Care Reporting System (NACRS).^14^ The Health Research Ethics Board of the University of Alberta (Pro00098444) approved this study. Alberta Health Services, the data custodians, provided operational and administrative approval. A data-sharing agreement governed data use. Informed consent was not obtained from individual patients, and only de-identified data were shared. No funding organization had any role in the conduct and reporting of this study.

### Study Setting and Population

We extracted data for all presentations to all EDs in Alberta, Canada from April 1, 2019–March 31, 2020. Alberta is a province in western Canada with 109 EDs that serve a population of 4.5 million people.^15^ Patients presenting with substance use disorder formed the study population. Presentations were classified as substance use if any one of the 10 diagnostic fields had International Statistical Classification of Diseases and Related Health Problems (ICD-10-CA)^16^ codes of mental and behavioral disorders due to psychoactive substance use (F10.0 to F19.9) or poisoning by drugs, medicaments, and biological substances (T36.0 to T50.9). These were the same codes used by Di Rico et al.^17^

### Study Protocol

The NACRS dataset included basic demographic information such as age, gender, and geography (postal code and health zone of residence). Dates/times of key points of the ED presentation (eg, start; physician initial assessment [PIA]; disposition decision), and ED facility are reported. Arrival mode (eg, ambulance), severity of a patient’s condition upon arrival based on the Canadian Triage and Acuity Scale (CTAS: 1 = resuscitation, 2 = emergent, 3 = urgent, 4 = less urgent, 5 = non-urgent),^18^ and up to 10 diagnosis fields using ICD-10-CA codes are provided. Presentations were categorized by substance type if any of the diagnosis fields included alcohol (any F10 [alcohol-related disorders]), MET use; any F15 [other stimulant-related disorders] or T43.6 [poisoning by, adverse effect of and underdosing of psychostimulants]), opioids (any F11 [opioid-related disorders] or T40 [poisoning by; adverse effect of; and underdosing of narcotics and psychodysleptics [hallucinogens]]), and other. Disposition included 16 categories that were grouped into discharged, admitted, transferred, left without being seen (LWBS), left against medical advice (LAMA), and died. There were few individuals who died in the ED, and they were excluded from the analyses.

**Population Health Research Capsule**

What do we already know about this issue?

*Substance use disorders are an increasingly common component of emergency department patient presentations with excessive resource utilization for their management*.

What was the research question?

*How does a diagnosis of substance use disorder affect a patient’s flow through the emergency department?*

What was the major finding of the study?

*When compared to other substances, methamphetamine use specifically caused a prolonged transition time from state to state*.

How does this improve population health?

*Emergency department flow may be improved by targeting interventions that expedite the care of patients with methamphetamine use disorder*.

We determined an indicator of ED crowding by calculating the median time from arrival to PIA for all presentations for any condition in each hour for each ED. Times over one hour were considered to be crowded in agreement with published guidelines.^19^

Linkages were made by Alberta Health Services to other Alberta Health databases to obtain additional variables. The Charlson Comorbidity Index^20^ based on a two-year lookback using NACRS and Discharge Abstract Database^21^ records was dichotomized into those without comorbidities (ie, 0) and those with at least one comorbidity (ie, ≥1). Using the Pampalon Index,^22^ we collapsed the average income of people aged >15 years into categories <$25,000, $25,000–$50,000, and >$50,000. And an urban status variable with four categories that were collapsed into metropolitan, urban, rural, and rural remote were provided based on local geographic boundaries of the patient’s residence.

### Key Outcome Measures

All patients begin in the start state. We determined the time associated with this as the minimum of the triage time and the time the patient presented to the ED. If a patient decides to leave prior to being seen by a physician, they are considered LWBS, in which case the time for transition is the time associated with patient departure from the ED. Once a patient is seen by a physician, they move to the PIA state, for which an exact time is recorded. From the PIA state, patients may move to the discharge disposition state, or the physician may decide to admit or transfer the patient instead, in which case they move to the admit/transfer disposition decision state. The time for both these transitions is given by the time to disposition. For patients whose ED presentation ends in LAMA, the time is given by the time associated with patient departure from the ED. If the decision is made to admit or transfer the patient, patients must wait again before transitioning into the admitted or transferred states. In either case, the time for this transition is given by the time of patient departure from the ED.

### Data Analysis

**Figure 1:**
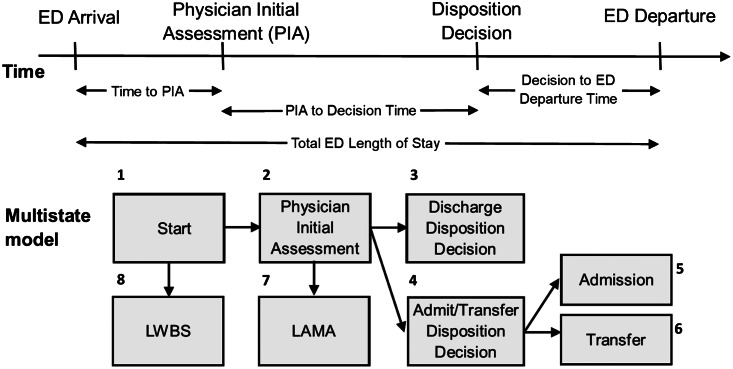
Multistate model of transitions through the emergency department. *LWBS*, left without being seen; *LAMA*, left against medical advice.

Summary statistics such as counts, percentages, medians, and interquartile ranges (IQR) represented as 25^th^ percentile, 75^th^ percentile) describe the characteristics of ED presentations. We used a multistate modelling framework to model the flow through the ED by considering eight mutually exclusive states as depicted in Figure 1: start; PIA; discharge disposition decision; admit/transfer disposition decision; admitted; transferred; LWBS; and LAMA. Initial models considered one covariate at a time to provide unadjusted hazard ratios (HR). Full models used all covariates, and these models were subsequently reduced via backwards selection until maximum parsimony was achieved (as per Akaike’s information criterion^23^). For all transitions, the covariates considered were gender, age, comorbidity, income category, weekend indicator (Saturday and Sunday were grouped as “weekend”), shift (day: 0800–1559, evening: 1600–2359, night: 0000–0759), arrival by ambulance, urban status, CTAS, indicators for alcohol, MET, and opioid use diagnoses, and the PIA crowding indicator.

We completed analyses in the statistical software R (R Foundation for Statistical Computing, Vienna, Austria).^24^ The msm package^25^ was used to fit all models, and the forest plot package^26^ displays HRs along with 95% confidence intervals. A HR >1 indicates that the time to transition is shorter for individuals of that group compared to the reference group, and a HR <1 indicates that the time to transition is longer for individuals of that group compared to the reference group.

## RESULTS

### Characteristics of ED Presentations

During the study period, there were 2,302,147 presentations to Alberta EDs, and 66,880 presentations were for substance use (37,530 patients) and available for analysis (Figure S1). The majority of patients had repeated presentations (78%), while only 22% presented to the ED once. The majority of presentations were by males (61.2%, Table 1), from the metropolitan areas (61.5%) in the health zones of Edmonton (36.1%) and Calgary (32.6%), and from neighborhoods where the average income was at least $25,000 (41.9% with $25,000–$50,000, 43.4% with >$50,000). Weekends were popular days for presentations (69.3%), and presentations were mostly considered urgent (CTAS 3 42.9%) or emergency (CTAS 2 37.5%). Based on the prior two years of ED and hospitalization data, comorbidities were not present for the vast majority of presentations (73.5%), and alcohol was the most common substance at the presentation (49.0%). Almost 65% of presentations ended in discharge, 19.3% ended in admission, and 7.4% ended in transfer.

**Figure 2:**
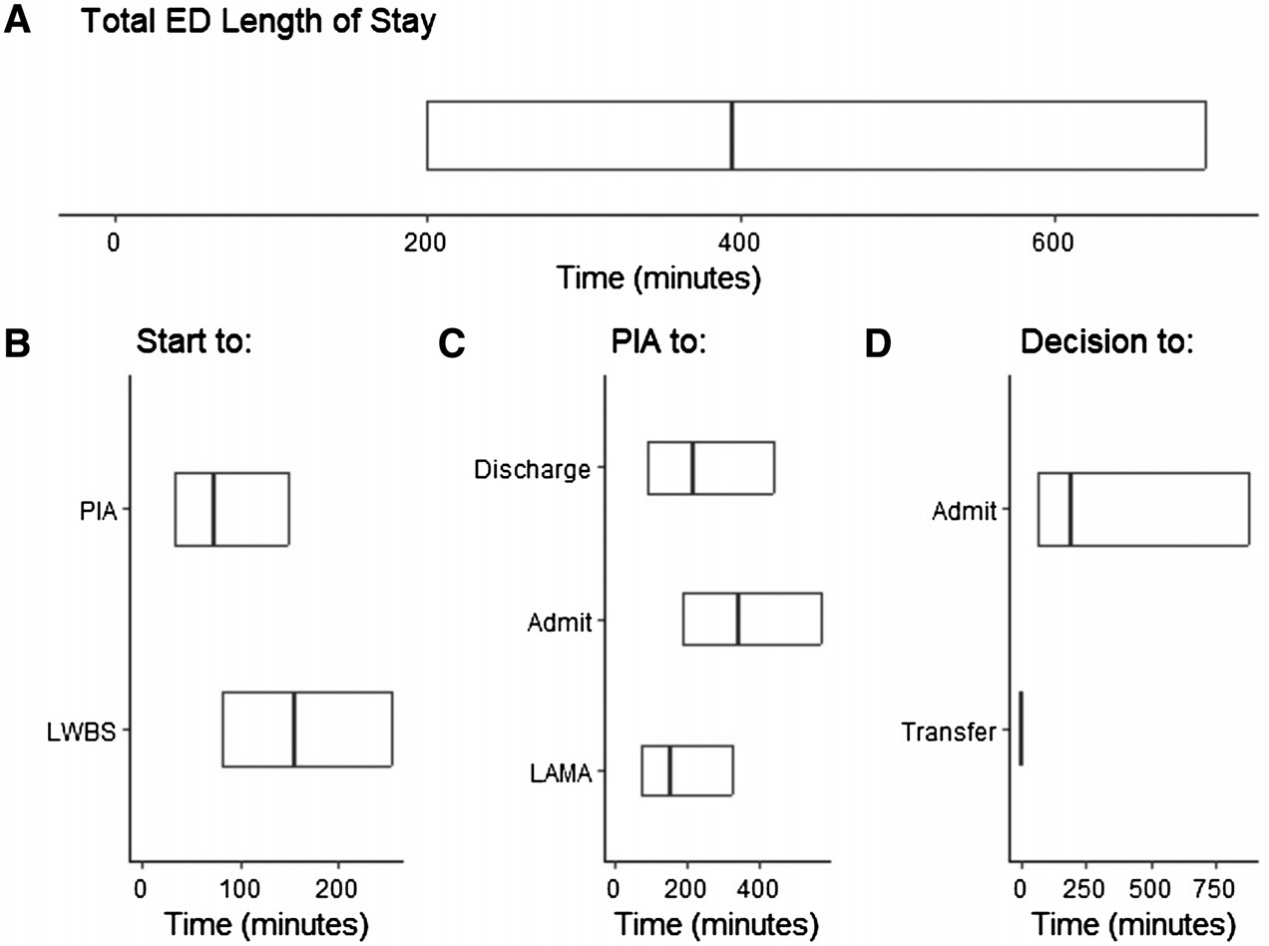
Summaries (25^th^ percentile, median, 75^th^ percentile) of the total length of stay (A) and the time spent in each state based on starting state: (B) start, (C) physician initial assessment (PIA), and (D) disposition decision. *ED*, emergency department.

Overall, the median time spent in the ED was 6 hours (h) 13 minutes (min) (IQR 3h 20min, 11h 36min, Table 2, Figure 2). Patients were seen by a physician in a median time of 1h 13min (IQR 32min, 2h 29min), and once seen, discharged patients had a median time to discharge of 3h 36min (IQR 1h 32min, 7h 20min) and admitted patients took longer with a median time to admission of 5h 39min (IQR 3h 8min, 9h 31min). We note that because most of the 4,204 patients who were transferred (State 6) had an instantaneous transition from the disposition decision state (State 4), the key summary statistics are 0h.

### Multistate Modelling

We focused on a few transitions using the multivariable model and have provided all results for unadjusted HRs and adjusted HRs in Tables S1 and S2, respectively, with forest plots for adjusted HRs in Figures S2–S4.

**Figure 3:**
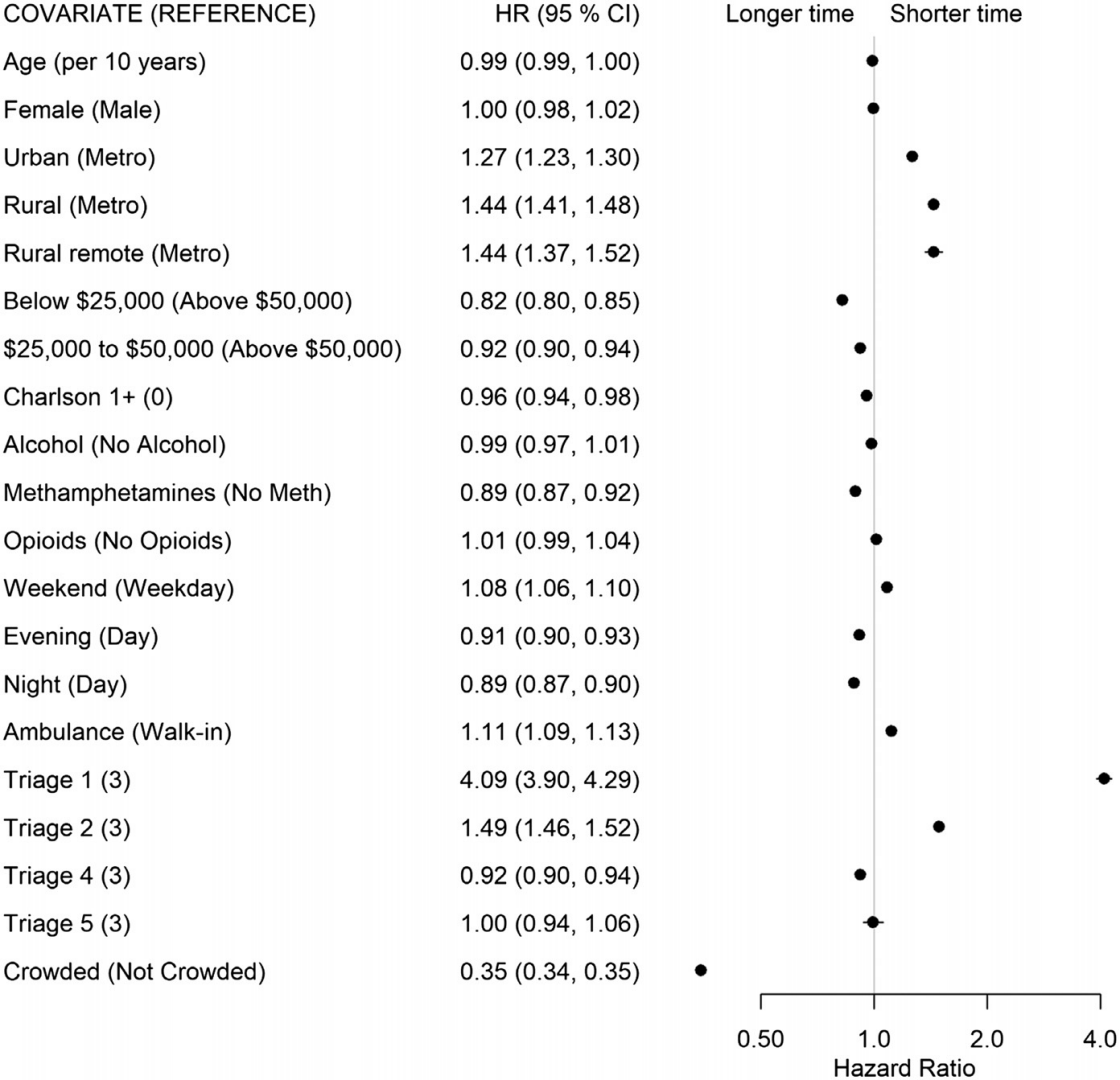
Forest plot of adjusted hazard ratios and associated 95% confidence intervals by covariates for the start (State 1) to physician initial assessment (State 2) transition for the multivariable model. *HR*, hazard ratio; *CI*, confidence interval.

For the start to PIA transition (State 1 to State 2), patients residing in urban (HR 1.27), rural (HR 1.44), and rural remote (HR 1.44) municipalities saw the physician quicker than patients from metropolitan municipalities. Patients with SUD were significantly affected by crowding. as patients in crowded EDs had longer times to wait to see a physician than patients presenting to uncrowded EDs (HR 0.35, Figure 3). Patients presenting with METH use also had longer times than those without METH use (HR 0.89). Lastly, patients from the lowest income neighborhoods had a longer transition time to PIA than those from neighborhoods with average incomes >$50,000 (HR 0.82).

**Figure 4:**
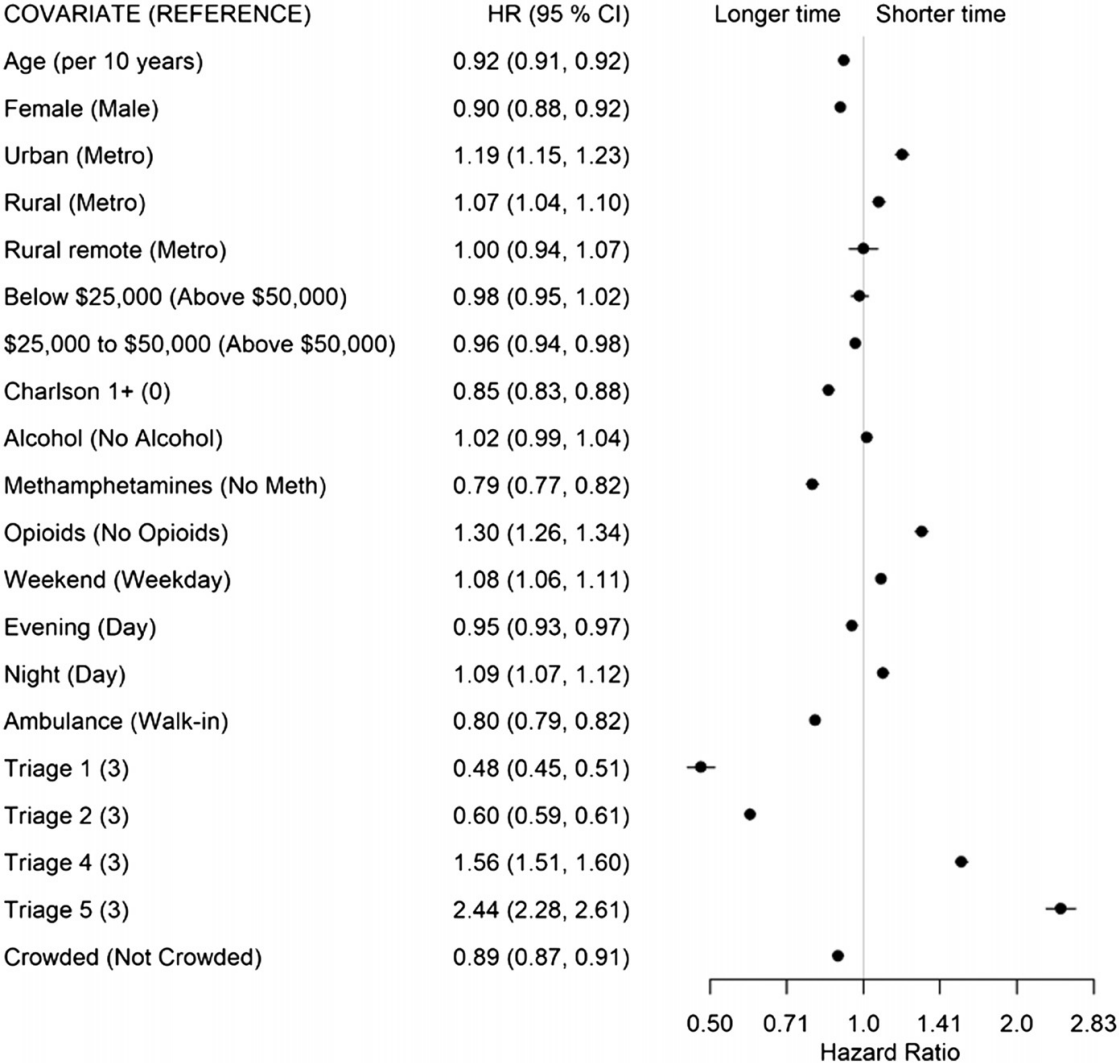
Forest plot of adjusted hazard ratios and associated 95% confidence intervals by covariates for the physician initial assessment (State 2) to discharge disposition decision (State 3) transition for the multivariable model. *HR*, hazard ratio; *CI*, confidence interval.

Once seen by a physician, lower acuity (CTAS 4:HR1.56, CTAS 5: HR 2.44) and opioid-related concerns (HR 1.30) had shorter times from PIA to discharge disposition decision (State 2 to 3, Figure 4). Longer times were associated with arriving by ambulance (HR 0.80), presenting with METH use (HR 0.79), with at least one comorbidity (HR 0.85), being female (HR 0.90), and older age (HR 0.92 per 10 years).

**Figure 5:**
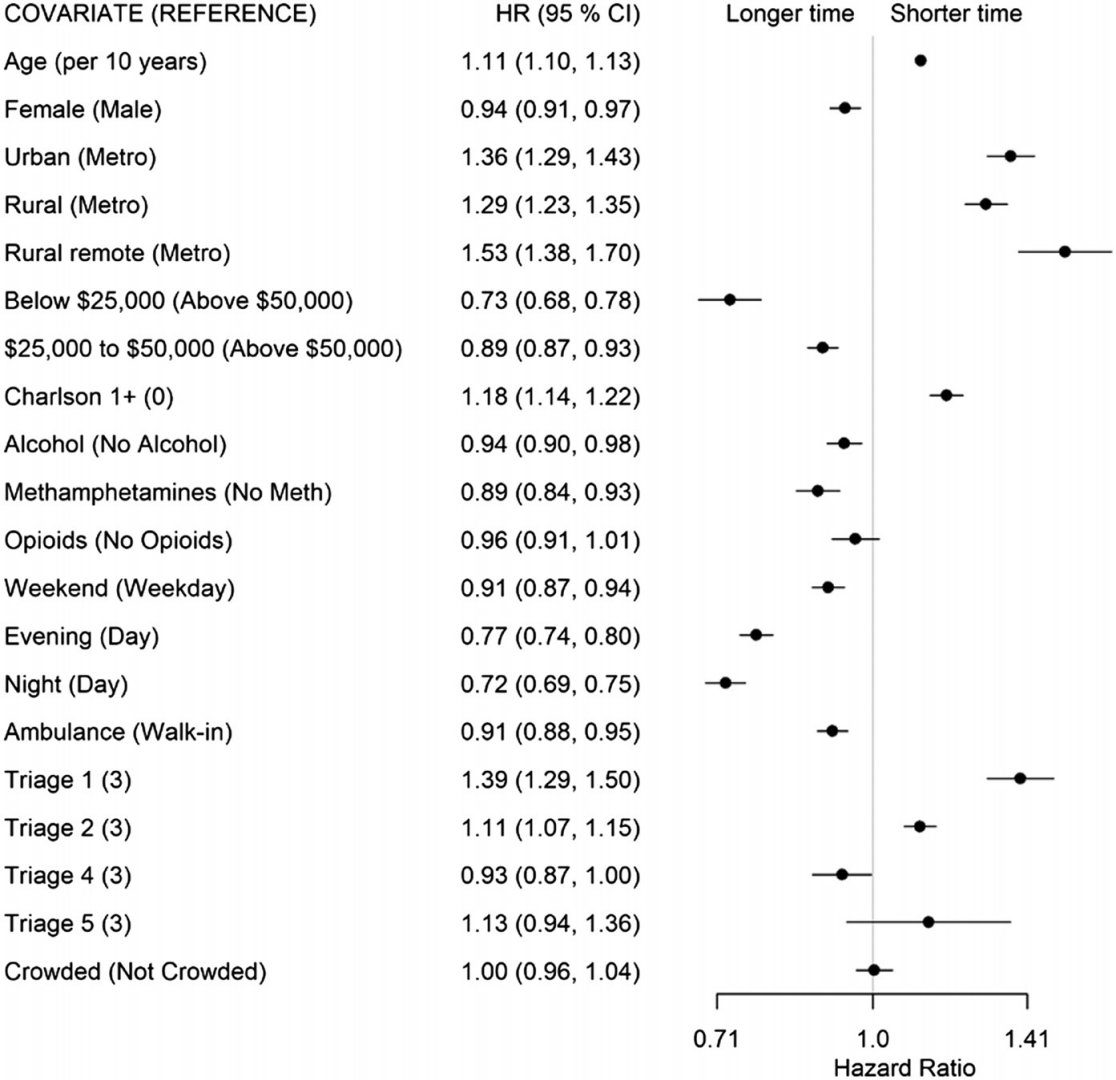
Forest plot of adjusted hazard ratios and associated 95% confidence intervals by covariates for the physician initial assessment (State 2) to admit/transfer disposition decision (State 4) transition for the multivariable model. *HR*, hazard ratio; *CI*, confidence interval.

For patients whose disposition decision was admission or transfer (State 2 to 4), higher acuity (CTAS 1: HR 1.39; CTAS 2: HR 1.11), living in a non-metropolitan municipality (urban: HR 1.36; rural: HR 1.29; remote: HR 1.53), having at least one comorbidity (HR 1.18), and older age (HR 1.11 per 10 years) had shorter transition times (Figure 5). Living in a neighborhood with low average income (<$25,000, HR 0.73), arriving after 4 pm (evening shift: HR 0.7; night shift: HR 0.72), arriving on a weekend (HR 0.91), arriving by ambulance (HR 0.91), and presenting with alcohol (HR 0.94) or METH use (HR 0.89) was associated with longer transition times. There was no evidence of ED crowding affecting this transition.

**Figure 6:**
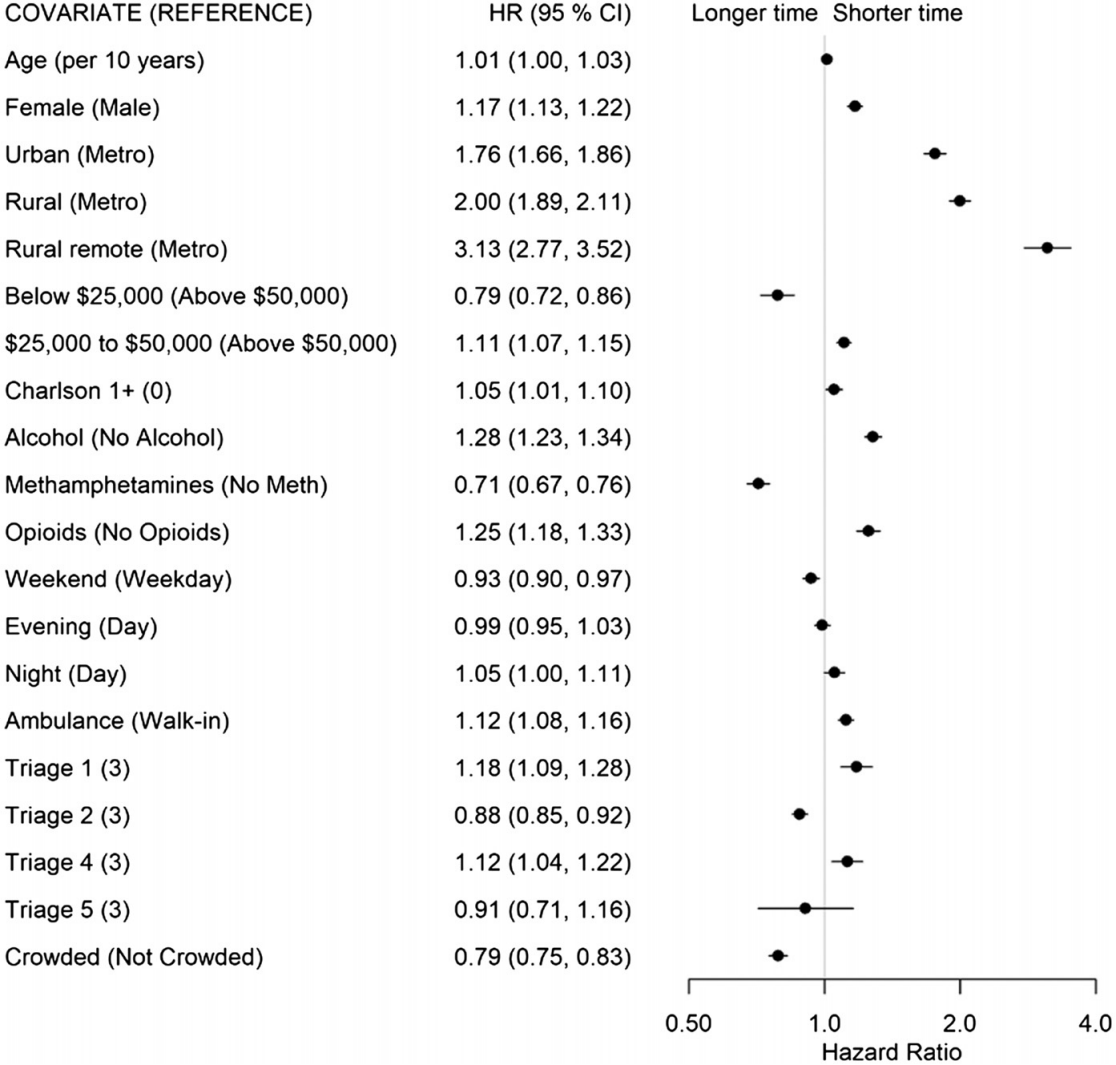
Forest plot of adjusted hazard ratios and associated 95% confidence intervals by covariates for the admit/transfer disposition decision (State 4) to admission (State 5) transition for the multivariable model. *HR*, hazard ratio; *CI*, confidence interval.

The factors associated with a shorter time for patients to be admitted out of the ED (State 4 to 5, Figure 6) included living in a non-metropolitan municipality (urban: HR 1.76; rural: H 2.00; remote: HR 3.13), presenting with opioid- (HR 1.25) or alcohol-related concerns (HR 1.28), and being female (HR 1.17). Conversely, living in a neighborhood with a low average income (<$25,000: HR 0.79), presenting with METH-related concerns (HR 0.71), and presenting to a crowded ED (HR 0.79) were associated with longer times to admission.

## DISCUSSION

In this population-based study we used over 37,000 patients with over 66,000 ED presentations to examine the flow through the ED for those presenting with SUD. The median LOS of patients included in our study (6h 13m) is comparable to the median LOS for all-cause patients presenting to major EDs in Alberta as reported by the provincial visual analytics platform Tableau (Tableau LLC, Seattle, WA) during the same time period (6h 18min).^27^ The total LOS was deconstructed into an eight-state multistate model, and there were different transition-specific effects for the explanatory variables. The transition to PIA time is one often used as a benchmark to a well functioning ED.^19^ Within this detailed look at SUD presentations to the ED, it is apparent that crowding particularly affects the SUD patient’s PIA time. Additionally, patients from neighborhoods with the lowest median income have longer transition times to their PIA, a result that echoes a prior study examining wait times in the unhoused population.^28^ A previous study^29^ identified that young adults experiencing homelessness have longer total ED stays, and the data presented here may highlight one factor for this in a SUD population. A theme that became apparent with this first state transition was that METH use was associated with prolonged time in the ED. The first state transition to PIA showed a modest increase in transition time for METH use. When compounded across all state transitions however, this particular SUD presentation creates significant delays in ED flow.

The decision to discharge a patient was the second state transition analyzed and showed a predictable effect among those SUD patients with low acuity. Of all SUD patients, those with opiate use presentation were able to transition to this state the fastest (HR 1.30). With the availability of a rapid reversal agent for opiates (naloxone), the medical management of opiate intoxication can potentially be completed within minutes^30^ and the patient can potentially be discharged within 1–2 hours. Subsequent monitoring and addressing the underlying opiate use disorder often make up the bulk of this patient’s ED LOS.^31^ The effect of gender, age, and mode of arrival have previously been identified to increase a patient’s ED LOS,^32^ but the data presented here show that this difference may be primarily influenced by the time to physician’s decision to discharge.

The effect of the Charlson Comorbidity Index on prolonging this specific transition between states is novel to our study. Previous studies addressing ED LOS have found no significant effect of a patient’s disease burden on their LOS; however, these studies were performed in countries outside of North America, where non-emergency physicians may be the first treating physician and the analyses were instead focused on factors that extended the total ED LOS.^33^,^34^ Like the previous transition analysis, METH use was associated with a prolonged decision to discharge. This could potentially be due to the clinical course of METH intoxication, where chemical restraints are more often required,^35^ and the physiological effects of METH can last more than 12 hours.^36^

The emergency physician’s decision to consult an admitting service for the patient was the third transition state analyzed. In EDs without an appropriate admitting service, this instead reflected the decision to transfer the patient out of the ED to a larger center that could accommodate the patient’s admission. Unsurprisingly, all those patients who had a higher acuity, additional comorbidities, or were older had this transition completed more quickly. All non-metropolitan EDs were also able to make this transition more quickly. This could be due to the physician’s faster recognition of a patient who cannot be managed at their smaller center and requires transfer, or a better relationship between the ED and inpatient units, which facilitates improved understanding of patients who would benefit from admission.

Those factors prolonging a decision to admit a patient include patient presentation during the evening or weekend hours and patients who arrive by ambulance. Qualitative studies have previously touched on some of these items^37^,^38^ that highlight healthcare system impacts on patient disposition. Our study provides quantitative evidence that supports these findings in the SUD patient population. Like the transition times for a decision to discharge, those patients with alcohol or METH use disorder also exhibited prolonged time-to-admission decisions. This is likely due to the period of observation common to both presentations to determine whether their symptoms will require additional support in the form of inpatient alcohol withdrawal treatments^39^ or further psychiatric assessment of the METH-use patient.^40^ Lastly, those patients from the lowest income bracket (which encompasses patients experiencing homelessness) experienced significantly longer transition times between these states.

The final state transition—that of a decision to admit/transfer the patient and their departure from the ED— showed a dramatically faster transition time in the non-metro EDs. This is likely due to similar factors that influenced the previous state change, namely the smaller EDs’ relationship with their admitting services or the ability to transfer the patient to another site for further management. Additionally, those patients presenting with either alcohol or opiate use were admitted much more quickly. Both presentations have well defined, nearly algorithmic management strategies consisting of alcohol withdrawal management in the form of benzodiazepines,^41^ and opiate intoxication management with naloxone. The association of METH use with a longer time to admission may be due to the management uncertainty surrounding it.^42^ Patients with METH use disorder can be behaviorally difficult,^43^ exhibit aggressive or bizarre behaviors, and may be accompanied by more trauma^44^ when compared to other SUD presentations. Emergency department crowding also negatively impacted a SUD patient’s transition to the admitted state, a finding that has been echoed over numerous studies of ED crowding.^45^,^46^ Lastly, patients from areas with the lowest median income once again spent more time in this transitory state. This patient cohort has been identified to be most at risk for poor clinical outcomes^47^,^48^ following an ED presentation and at highest risk for leaving AMA.^49^ The extended delays between numerous state transitions serve to highlight some of the shortcomings of both the ED and admitting teams in their care.

## LIMITATIONS

Our study has limitations including those typical of data collected for administrative purposes. We used the times provided for each state transition and those times may have been reported in error or may have been missing. There may be other important variables that contributed to the times spent in each state that we were unable to account for in our analysis. These may include characteristics of the patient or characteristics of the ED, such as staffing, that are not available in the data sources.

## CONCLUSION

Taken together, this study demonstrated two pervasive themes across all state transitions. Patients who presented with methamphetamines use disorder had delayed transitions between all states analyzed during their ED visit. Similarly, patients from low-income neighborhoods also had delays in almost all transitions analyzed. With the data presented here, the emergency medicine community may benefit from improved ED flow by focusing on improving transition times for the low socioeconomic status patient with METH use disorder. Due to the challenges with managing METH intoxication, further research to improve treatment pathways for these patients will ultimately help ED flow as well. Additional resources may be required to assist the urban ED with managing the influx of these patients with substance use disorder and their overall effect on ED crowding.

## Supplementary Information



## Figures and Tables

**Table 1:** Characteristics of 67,416 emergeny department presentations for substance use.

	n	(%)
Age in years, mean (SD)	37.2	15.1
Gender		
Male	40,913	61.2
Female	25,961	38.8
Missing	6	0.0
Health Zone of Residence		
North	7,474	11.2
Edmonton	24,159	36.1
Central	6,510	9.7
Calgary	21,792	32.6
South	5,705	8.5
Missing	1,240	1.9
Population Centre Type of Residence		
Metro	41,119	61.5
Urban	7,256	10.8
Rural	12,379	18.5
Remote	1,816	2.7
Missing	4,310	6.4
Average Neighborhood Income		
< $25,000	5,550	8.3
$25,000–$50,000	28,020	41.9
> $50,000	29,000	43.4
Missing	4,310	6.4
Number of Comorbidities per the Charlson Comorbidity Index		
0	49,158	73.5
≥1	17,722	26.5
Arrival Mode		
Ambulance	32,731	48.9
Day of Week		
Weekday	46,319	69.3
Weekend	20,561	30.7
Time of Day		
Day (0800–1559)	21,194	31.7
Evening (1600–2359)	28,162	42.1
Night (0000–0759)	17,524	26.2
Triage Level		
1 = Resuscitation	2,222	3.3
2 = Emergent	25,063	37.5
3 = Urgent	28,671	42.9
4 = Less Urgent	9,131	13.7
5 = Non-urgent	1,236	1.8
Missing	557	0.8
Diagnostic Category		
Alcohol	32,780	49.0
Methamphetamines	10,759	16.1
Opioids	9,720	14.5
None of the above	16,837	25.2
Disposition		
Discharged	43,190	64.6
Admitted	12,921	19.3
Transferred	4,918	7.4
Left before being seen	3,652	5.5
Left against medical advice	2,199	3.3
Crowding Level		
Crowded (median time to physician initial assessment >1 hour)	48,149	72.0

*ED*, emergency department.

**Table 2:** Summaries of the time spent in each state.

Period	n	Median	(25^th^ percentile, 75^th^ percentile)
Total Length of Stay	66,880	6h 13min	3h 20min, 11h 36min
Start to Physician Initial Assessment (PIA) (State 1 to 2)	61,167	1h 13min	0h 32min, 2h 29min
PIA to Discharge Disposition (State 2 to 3)	43,189	3h 36min	1h 32min, 7h 20min
PIA to Admit or Transfer Disposition (State 2 to 4)	15,763	5h 39min	3h 8min, 9h 31min
Admit or Transfer Disposition to Admission (State 4 to 5)	12,906	3h 16min	1h 4min, 14h 32min
Admit or Transfer Disposition to Transfer (State 4 to 6)	4,204	0h 0min	0h 0min, 0h 0min
Start to Left Without Being Seen (State 1 to 8)	3,652	2h 34min	1h 21min, 4h 14min
PIA to Left Against Medical Advice (State 2 to 7)	1,417	2h 35min	1h 10min, 5h 25min

*h*, hours; *min*, minutes.
