# Supplementary material for: Flow through the Emergency Department for Patients Presenting with Substance Use Disorder in Alberta, Canada
Source: West J Emerg Med. 2023 Jul 7;24(4):717–27. doi: 10.5811/westjem.60350 (PMC10393443; doi:10.5811/westjem.60350)
Supplement: Supplementary file 1 [file wjem-24-717-s001.docx]

Figure S1: Flow diagram of patients included in multi-state modeling.


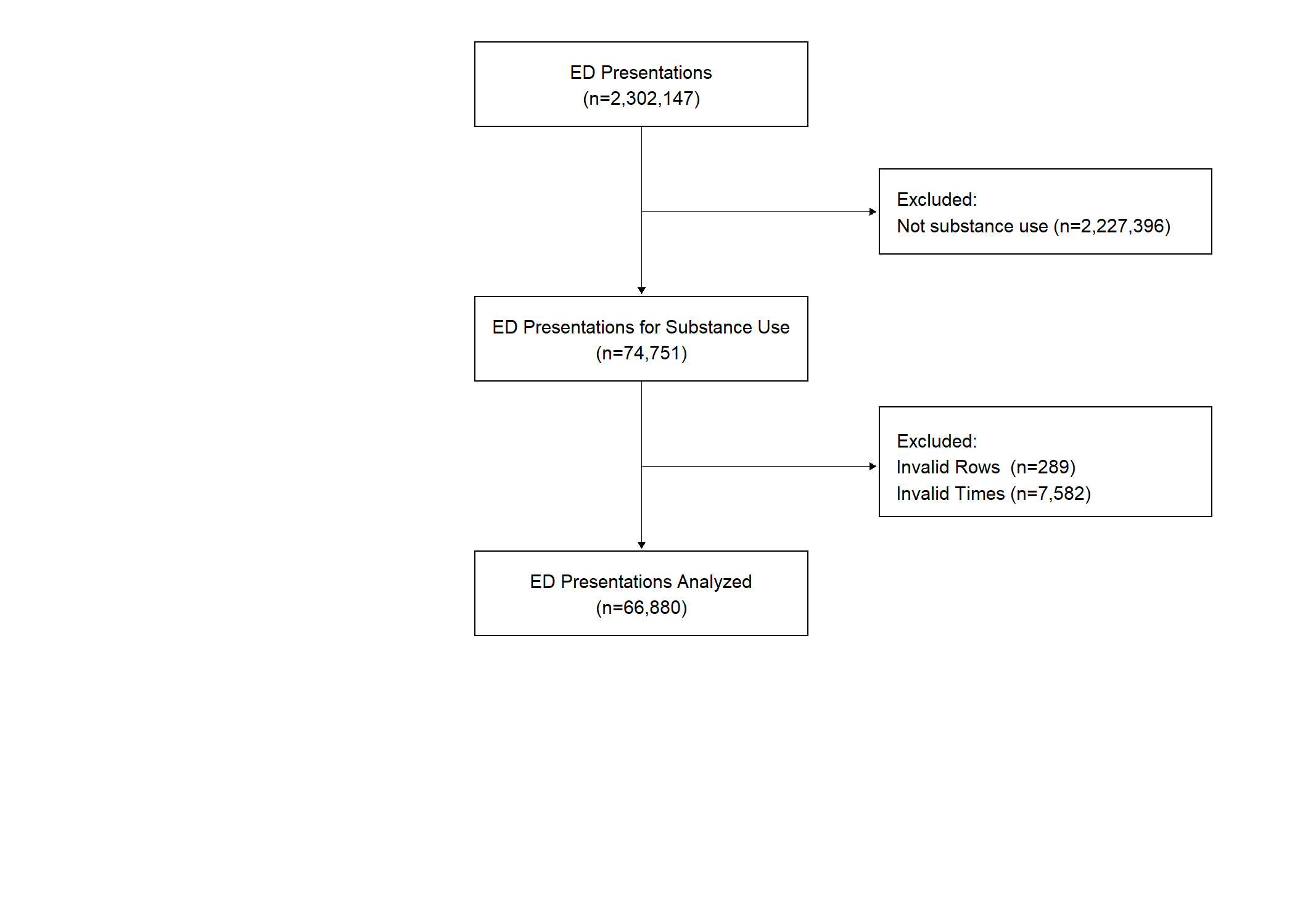


Figure S2. Forest plot of adjusted hazard ratios (HRs) and associated 95% confidence intervals (CIs) by covariates for the start (State 1) to left without being seen (State 8) transition for the multivariable model.


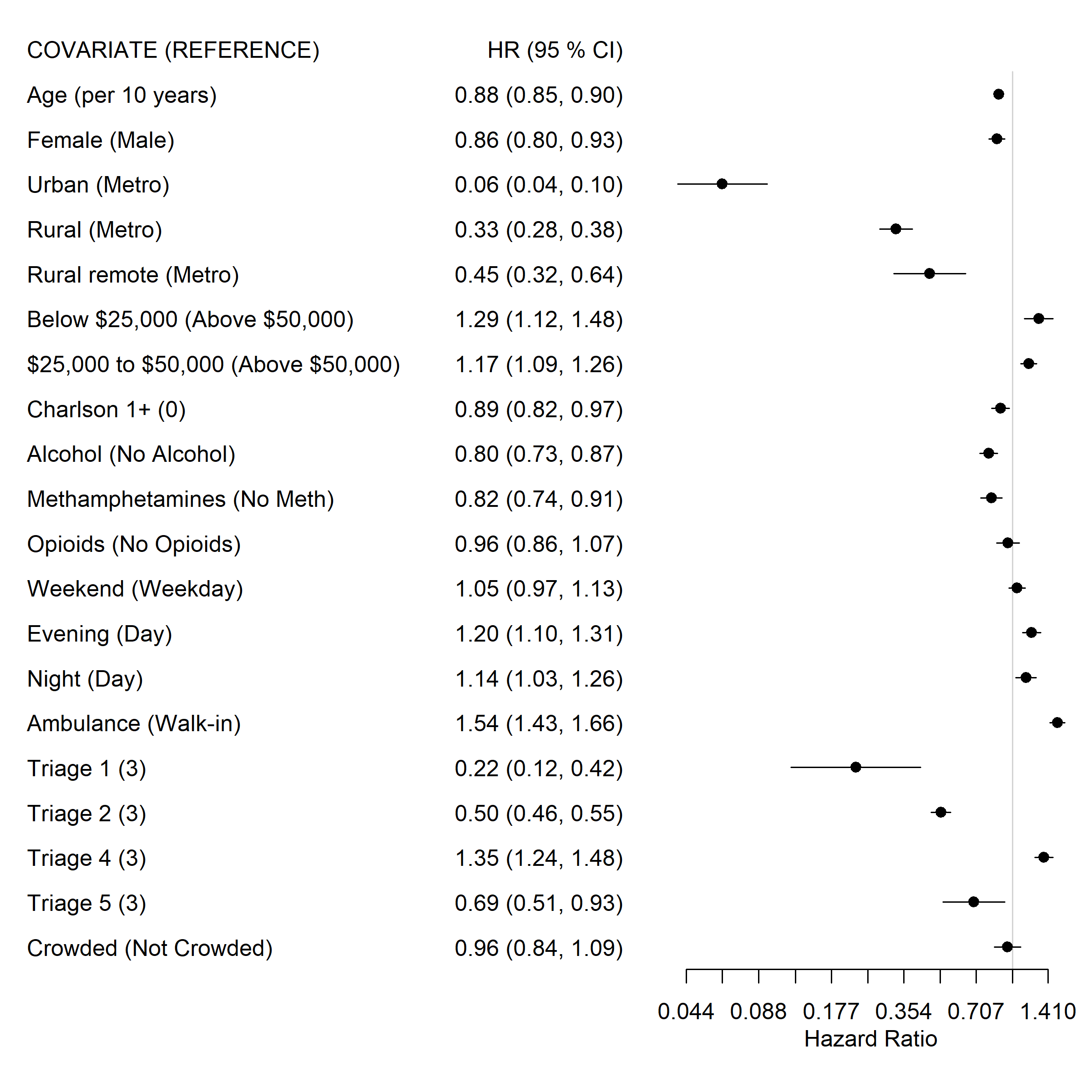


Figure S3. Forest plot of adjusted hazard ratios (HRs) and associated 95% confidence intervals (CIs) by covariates for the physician initial assessment (State 2) to left against medical advice (State 7) transition for the multivariable model.


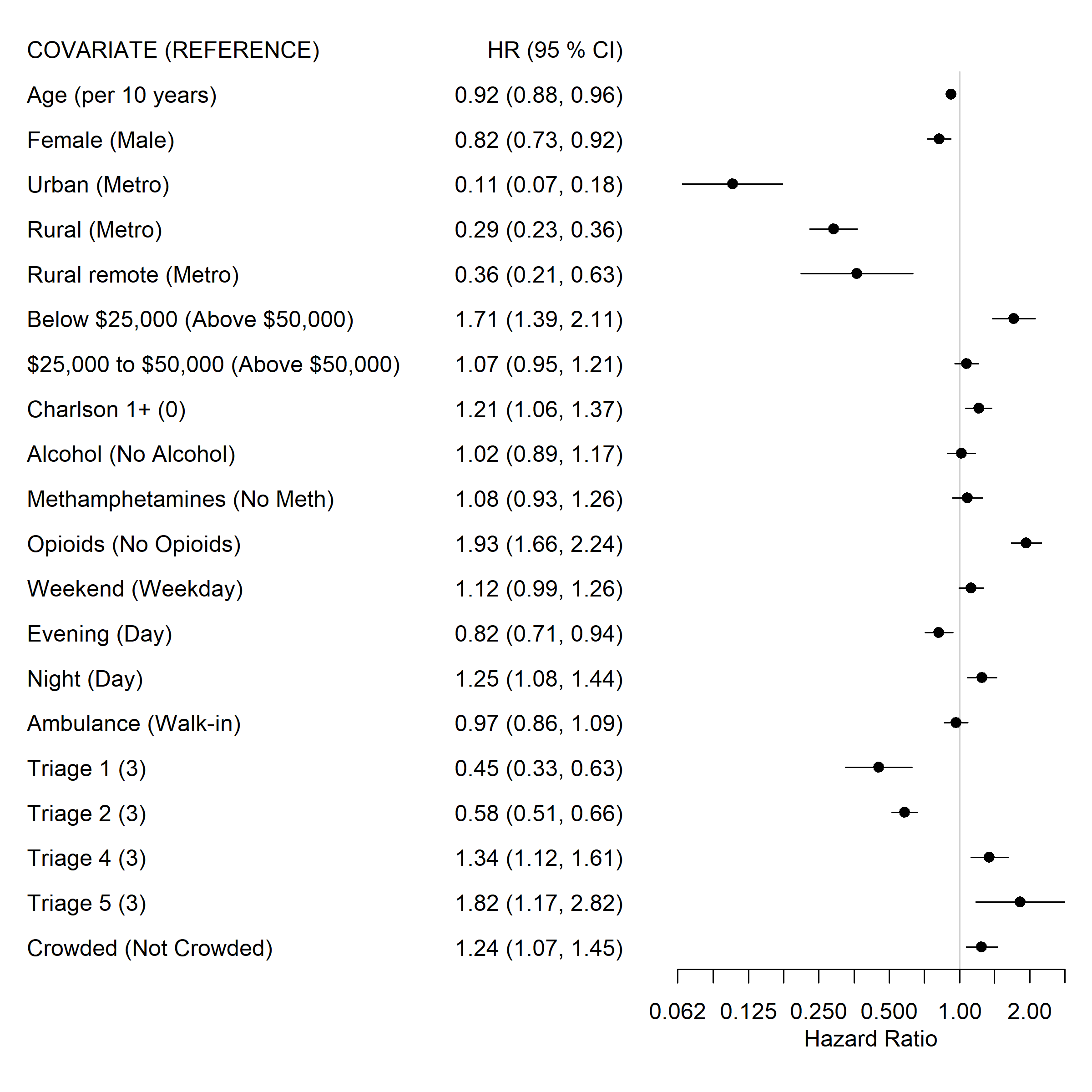


Figure S4. Forest plot of adjusted hazard ratios (HRs) and associated 95% confidence intervals (CIs) by covariates for the admit/transfer disposition decision (State 4) to departure (State 6) transition for the multivariable model.


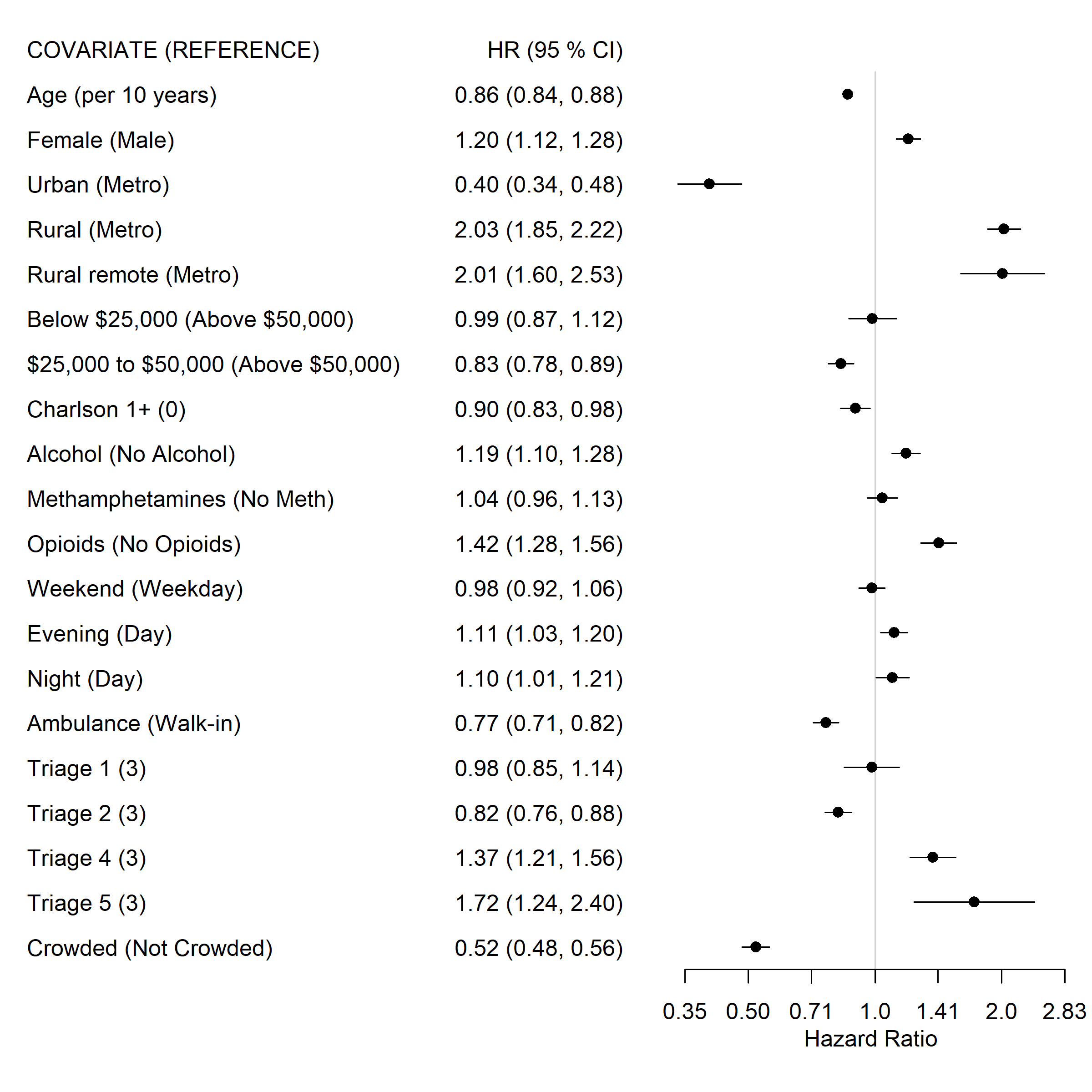


Figure S5. Kaplan-Meier plot for the time from start (State 1) to physician initial assessment (State 2) by ED crowding indicator (Crowded if median time from arrival to physician initial assessment for all presentations for any condition in during the same hour and same ED was >1 hour).
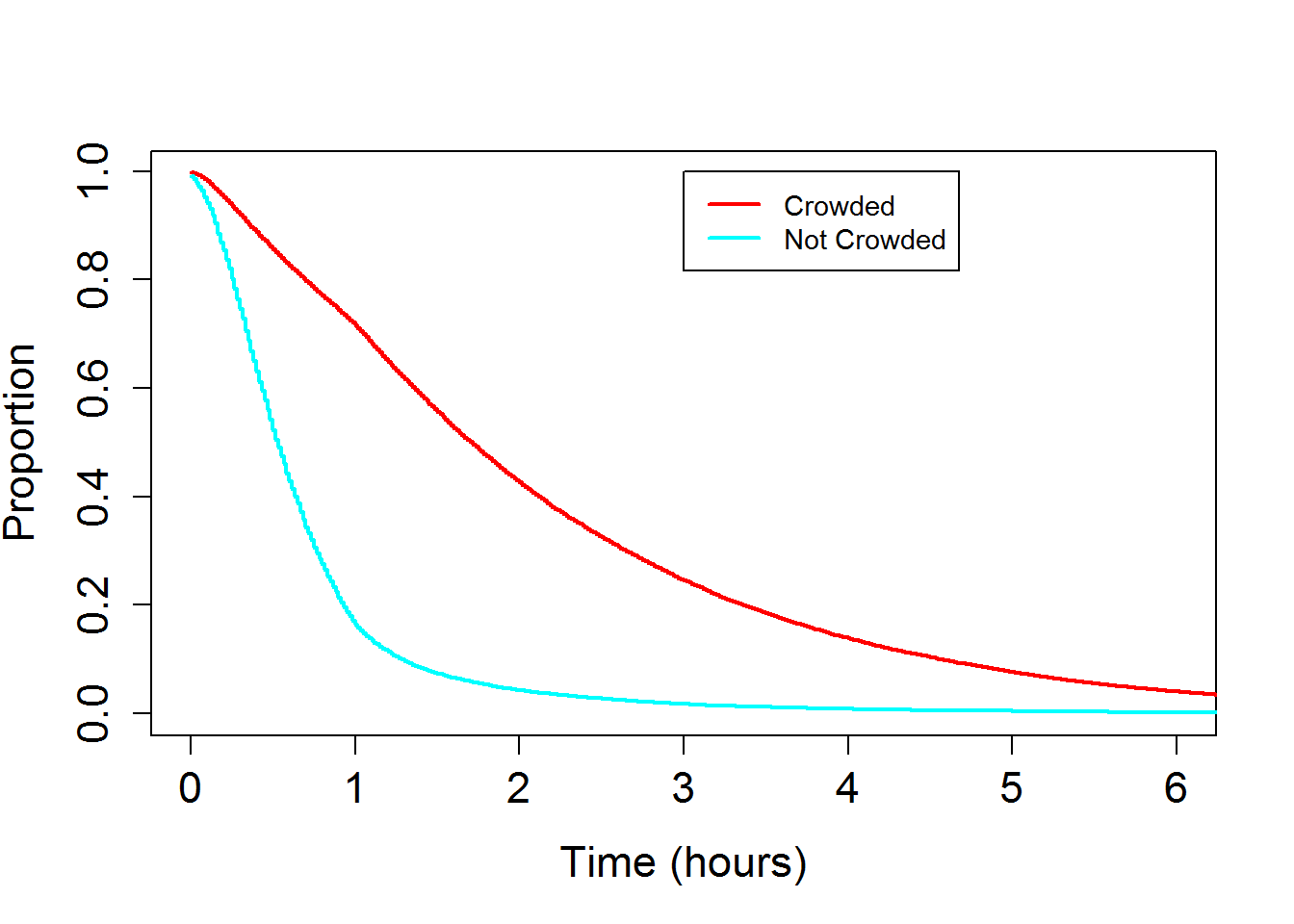


Table S1. Unadjusted hazard ratios (HRs) and associated 95% confidence intervals (CIs) for each state transition. States are: 1 Start, 2 Physician Initial Assessment, 3 Discharge Disposition Decision, 4 Admit/Transfer Disposition Decision, 5 Admission, 6 Departure, 7 Left Against Medical Advice, 8 Left Without Being Seen.

|  |  | Transition | | | | | | | | | | | | | |
| --- | --- | --- | --- | --- | --- | --- | --- | --- | --- | --- | --- | --- | --- | --- | --- |
|  |  | 1 to 2 | | 1 to 8 | | 2 to 3 | | 2 to 4 | | 2 to 7 | | 4 to 5 | | 4 to 6 | |
|  |  | HR | 95% CI | HR | 95% CI | HR | 95% CI | HR | 95% CI | HR | 95% CI | HR | 95% CI | HR | 95% CI |
| Age, per 10 years | | 0.96 | 0.96, 0.97* | 0.93 | 0.91, 0.95* | 0.92 | 0.91, 0.93* | 1.13 | 1.11, 1.14* | 0.97 | 0.93, 1.00 | 1.07 | 1.06, 1.08* | 0.85 | 0.83, 0.87* |
| Sex | |  |  |  |  |  |  |  |  |  |  |  |  |  |  |
|  | Male | Ref |  |  |  |  |  |  |  |  |  |  |  |  |  |
|  | Female | 1.08 | 1.06, 1.09* | 0.86 | 0.81, 0.93* | 0.91 | 0.90, 0.93* | 0.92 | 0.89, 0.95* | 0.81 | 0.73, 0.90* | 1.14 | 1.10, 1.18* | 1.19 | 1.12, 1.27* |
| Urban Status | |  |  |  |  |  |  |  |  |  |  |  |  |  |  |
|  | Metro | Ref |  |  |  |  |  |  |  |  |  |  |  |  |  |
|  | Urban | 1.65 | 1.61, 1.69* | 0.07 | 0.05, 0.11* | 1.40 | 1.36, 1.44* | 1.31 | 1.25, 1.38* | 0.11 | 0.07, 0.18* | 1.98 | 1.88, 2.09* | 0.53 | 0.45, 0.62* |
|  | Rural | 1.66 | 1.63, 1.70* | 0.38 | 0.32, 0.43* | 1.25 | 1.22, 1.28* | 1.18 | 1.13, 1.23* | 0.34 | 0.27, 0.42* | 2.40 | 2.30, 2.51* | 2.70 | 2.51, 2.90* |
|  | Remote | 1.66 | 1.57, 1.75* | 0.50 | 0.36, 0.70* | 1.31 | 1.23, 1.40* | 1.38 | 1.25, 1.53* | 0.41 | 0.24, 0.70* | 5.29 | 4.83, 5.79* | 3.64 | 3.03, 4.37* |
| Average Neighbourhood Income | |  |  |  |  |  |  |  |  |  |  |  |  |  |  |
|  | < $25,000 | 0.93 | 0.90, 0.96* | 1.04 | 0.92, 1.19 | 1.07 | 1.03, 1.11* | 0.83 | 0.78, 0.88* | 1.18 | 0.97, 1.44 | 1.41 | 1.31, 1.51* | 1.67 | 1.50, 1.87* |
|  | $25,000-$50,000 | 0.93 | 0.91, 0.94* | 1.13 | 1.05, 1.21* | 0.98 | 0.96, 1.00* | 0.91 | 0.88, 0.95* | 1.02 | 0.91, 1.15 | 1.17 | 1.13, 1.22* | 0.87 | 0.81, 0.93* |
|  | > $50,000 | Ref |  |  |  |  |  |  |  |  |  |  |  |  |  |
| Comorbidities | |  |  |  |  |  |  |  |  |  |  |  |  |  |  |
|  | No | Ref |  |  |  |  |  |  |  |  |  |  |  |  |  |
|  | Yes | 0.87 | 0.85, 0.88* | 0.93 | 0.87, 1.00 | 0.81 | 0.79, 0.83* | 1.28 | 1.24, 1.32* | 1.29 | 1.16, 1.44* | 1.25 | 1.21, 1.30* | 0.82 | 0.76, 0.88* |
| Day of Week | |  |  |  |  |  |  |  |  |  |  |  |  |  |  |
|  | Weekday | Ref |  |  |  |  |  |  |  |  |  |  |  |  |  |
|  | Weekend | 1.12 | 1.10, 1.14* | 1.06 | 0.99, 1.14 | 1.10 | 1.07, 1.12* | 0.88 | 0.85, 0.92* | 1.11 | 0.99, 1.24 | 0.96 | 0.92, 0.99* | 1.03 | 0.96, 1.10 |
| Shift | |  |  |  |  |  |  |  |  |  |  |  |  |  |  |
|  | Day (0800 - 1559) | Ref |  |  |  |  |  |  |  |  |  |  |  |  |  |
|  | Evening (1600 - 2359) | 0.87 | 0.85, 0.88* | 1.23 | 1.14, 1.33* | 0.92 | 0.90, 0.94* | 0.76 | 0.74, 0.79* | 0.79 | 0.70, 0.90* | 0.97 | 0.93, 1.01 | 1.14 | 1.06, 1.22* |
|  | Night (0000 - 0759) | 0.88 | 0.86, 0.90* | 1.24 | 1.13, 1.35* | 1.14 | 1.11, 1.16* | 0.68 | 0.65, 0.71* | 1.28 | 1.22, 1.46* | 1.08 | 1.03, 1.13* | 1.22 | 1.13, 1.33* |
| Arrival Mode | |  |  |  |  |  |  |  |  |  |  |  |  |  |  |
|  | Walk-in | Ref |  |  |  |  |  |  |  |  |  |  |  |  |  |
|  | Ambulance | 1.15 | 1.14, 1.17* | 1.35 | 1.27, 1.45* | 0.72 | 0.71, 0.74* | 0.96 | 0.93, 0.99* | 0.90 | 0.81, 1.00* | 1.23 | 1.19, 1.27* | 0.79 | 0.74, 0.84* |
| Triage Level | |  |  |  |  |  |  |  |  |  |  |  |  |  |  |
|  | 1=Resuscitation | 4.22 | 4.04, 4.42* | 0.31 | 0.18, 0.54* | 0.48 | 0.45, 0.51* | 1.23 | 1.15, 1.32* | 0.51 | 0.37, 0.70* | 1.05 | 0.97, 1.13 | 1.09 | 0.95, 1.25 |
|  | 2=Emergent | 1.52 | 1.50, 1.55* | 0.56 | 0.51, 0.61* | 0.61 | 0.60, 0.63* | 1.03 | 0.99, 1.06 | 0.60 | 0.54, 0.68* | 0.77 | 0.74, 0.80* | 0.83 | 0.77, 0.88* |
|  | 3=Urgent | Ref |  |  |  |  |  |  |  |  |  |  |  |  |  |
|  | 4=Less Urgent | 0.93 | 0.90, 0.95* | 1.24 | 1.15, 1.35* | 1.58 | 1.53, 1.62* | 0.95 | 0.89, 1.01 | 1.41 | 1.20, 1.65* | 1.11 | 1.03, 1.19* | 1.42 | 1.27, 1.60* |
|  | 5=Non-Urgent | 0.99 | 0.93, 1.05 | 0.65 | 0.50, 0.84* | 2.63 | 2.47, 2.80* | 1.12 | 0.94, 1.33 | 1.80 | 1.19, 2.73* | 1.00 | 0.80, 1.24 | 1.65 | 1.21, 2.25* |
| Diagnostic Category | |  |  |  |  |  |  |  |  |  |  |  |  |  |  |
|  | Alcohol | 0.98 | 0.96, 1.00* | 0.86 | 0.81, 0.92* | 1.00 | 0.98, 1.02 | 1.07 | 1.04, 1.10* | 0.86 | 0.77, 0.95* | 1.46 | 1.41, 1.51* | 0.96 | 0.90, 1.02 |
|  | Methamphetamines | 0.82 | 0.80, 0.84* | 1.03 | 0.95, 1.12 | 0.85 | 0.83, 0.88* | 0.80 | 0.76, 0.83* | 1.18 | 1.04, 1.34* | 0.59 | 0.56, 0.63* | 1.10 | 1.02, 1.18* |
|  | Opioids | 1.04 | 1.02, 1.06* | 1.11 | 1.02, 1.22* | 1.21 | 1.18, 1.24* | 1.00 | 0.95, 1.05 | 1.92 | 1.70, 2.18* | 1.19 | 1.13, 1.25* | 1.28 | 1.17, 1.40* |
| Crowding Level | |  |  |  |  |  |  |  |  |  |  |  |  |  |  |
|  | Not crowded | Ref |  |  |  |  |  |  |  |  |  |  |  |  |  |
|  | Crowded | 0.31 | 0.30, 0.31* | 1.30 | 1.16, 1.47* | 0.78 | 0.76, 0.79* | 0.93 | 0.89, 0.96* | 1.68 | 1.46, 1.94* | 0.62 | 0.60, 0.65* | 0.49 | 0.46, 0.53* |

Table S2. Adjusted hazard ratios (HRs) and associated 95% confidence intervals (CIs) for each state transition based on a multivariable model with all covariates. States are: 1 Start, 2 Physician Initial Assessment, 3 Discharge Disposition Decision, 4 Admit/Transfer Disposition Decision, 5 Admission, 6 Departure, 7 Left Against Medical Advice, 8 Left Without Being Seen.

|  |  | Transition | | | | | | | | | | | | | |
| --- | --- | --- | --- | --- | --- | --- | --- | --- | --- | --- | --- | --- | --- | --- | --- |
|  |  | 1 to 2 | | 1 to 8 | | 2 to 3 | | 2 to 4 | | 2 to 7 | | 4 to 5 | | 4 to 6 | |
|  |  | HR | 95% CI | HR | 95% CI | HR | 95% CI | HR | 95% CI | HR | 95% CI | HR | 95% CI | HR | 95% CI |
| Age, per 10 years | | 0.99 | 0.99, 1.00* | 0.88 | 0.85, 0.90* | 0.92 | 0.91, 0.92* | 1.11 | 1.10, 1.13* | 0.92 | 0.88, 0.96* | 1.01 | 1.00, 1.03* | 0.86 | 0.84, 0.88* |
| Sex | |  |  |  |  |  |  |  |  |  |  |  |  |  |  |
|  | Male | Ref |  |  |  |  |  |  |  |  |  |  |  |  |  |
|  | Female | 1.00 | 0.98, 1.02 | 0.86 | 0.80, 0.93* | 0.90 | 0.88, 0.92* | 0.94 | 0.91, 0.97* | 0.82 | 0.73, 0.92* | 1.17 | 1.13, 1.22* | 1.20 | 1.12, 1.28* |
| Urban Status | |  |  |  |  |  |  |  |  |  |  |  |  |  |  |
|  | Metro | Ref |  |  |  |  |  |  |  |  |  |  |  |  |  |
|  | Urban | 1.27 | 1.23, 1.30* | 0.06 | 0.04, 0.10* | 1.19 | 1.15, 1.23* | 1.36 | 1.29, 1.43* | 0.11 | 0.07, 0.18* | 1.76 | 1.66, 1.86* | 0.40 | 0.34, 0.48* |
|  | Rural | 1.44 | 1.41, 1.48* | 0.33 | 0.28, 0.38* | 1.07 | 1.04, 1.10* | 1.29 | 1.23, 1.35* | 0.29 | 0.23, 0.36* | 2.00 | 1.89, 2.11* | 2.03 | 1.85, 2.22* |
|  | Remote | 1.44 | 1.37, 1.52* | 0.45 | 0.32, 0.64* | 1.00 | 0.94, 1.07 | 1.53 | 1.38, 1.70* | 0.36 | 0.21, 0.63* | 3.13 | 2.77, 3.52* | 2.01 | 1.60, 2.53* |
| Average Neighbourhood Income | |  |  |  |  |  |  |  |  |  |  |  |  |  |  |
|  | < $25,000 | 0.82 | 0.80, 0.85* | 1.29 | 1.12, 1.48* | 0.98 | 0.95, 1.02 | 0.73 | 0.68, 0.78* | 1.71 | 1.39, 2.11* | 0.79 | 0.72, 0.86* | 0.99 | 0.87, 1.12 |
|  | $25,000-$50,000 | 0.92 | 0.90, 0.94* | 1.17 | 1.09, 1.26* | 0.96 | 0.94, 0.98* | 0.89 | 0.87, 0.93* | 1.07 | 0.95, 1.21 | 1.11 | 1.07, 1.15* | 0.83 | 0.78, 0.89* |
|  | > $50,000 | Ref |  |  |  |  |  |  |  |  |  |  |  |  |  |
| Comorbidities | |  |  |  |  |  |  |  |  |  |  |  |  |  |  |
|  | No | Ref |  |  |  |  |  |  |  |  |  |  |  |  |  |
|  | Yes | 0.96 | 0.94, 0.98* | 0.89 | 0.82, 0.97* | 0.85 | 0.83, 0.88* | 1.18 | 1.14, 1.22* | 1.21 | 1.06, 1.37* | 1.05 | 1.01, 1.10* | 0.90 | 0.83, 0.98* |
| Day of Week | |  |  |  |  |  |  |  |  |  |  |  |  |  |  |
|  | Weekday | Ref |  |  |  |  |  |  |  |  |  |  |  |  |  |
|  | Weekend | 1.08 | 1.06, 1.10* | 1.05 | 0.97, 1.13 | 1.08 | 1.06, 1.11* | 0.91 | 0.87, 0.94* | 1.12 | 0.99, 1.26 | 0.93 | 0.90, 0.97* | 0.98 | 0.92, 1.06 |
| Shift | |  |  |  |  |  |  |  |  |  |  |  |  |  |  |
|  | Day (0800 - 1559) | Ref |  |  |  |  |  |  |  |  |  |  |  |  |  |
|  | Evening (1600 - 2359) | 0.91 | 0.90, 0.93* | 1.20 | 1.10, 1.31* | 0.95 | 0.93, 0.97* | 0.77 | 0.74, 0.80* | 0.82 | 0.71, 0.94* | 0.99 | 0.95, 1.03 | 1.11 | 1.03, 1.20* |
|  | Night (0000 - 0759) | 0.89 | 0.87, 0.90* | 1.14 | 1.03, 1.26* | 1.09 | 1.07, 1.12* | 0.72 | 0.69, 0.75* | 1.25 | 1.08, 1.44* | 1.05 | 1.00, 1.11 | 1.10 | 1.01, 1.21* |
| Arrival Mode | |  |  |  |  |  |  |  |  |  |  |  |  |  |  |
|  | Walk-in | Ref |  |  |  |  |  |  |  |  |  |  |  |  |  |
|  | Ambulance | 1.11 | 1.09, 1.13* | 1.54 | 1.43, 1.66* | 0.80 | 0.79, 0.82* | 0.91 | 0.88, 0.95* | 0.97 | 0.86, 1.09 | 1.12 | 1.08, 1.16* | 0.77 | 0.71, 0.82* |
| Triage Level | |  |  |  |  |  |  |  |  |  |  |  |  |  |  |
|  | 1=Resuscitation | 4.09 | 3.90, 4.29* | 0.22 | 0.12, 0.42* | 0.48 | 0.45, 0.51* | 1.39 | 1.29, 1.50* | 0.45 | 0.33, 0.63* | 1.18 | 1.09, 1.28* | 0.98 | 0.85, 1.14 |
|  | 2=Emergent | 1.49 | 1.46, 1.52* | 0.50 | 0.46, 0.55* | 0.60 | 0.59, 0.61* | 1.11 | 1.07, 1.15* | 0.58 | 0.51, 0.66* | 0.88 | 0.85, 0.92* | 0.82 | 0.76, 0.88* |
|  | 3=Urgent | Ref |  |  |  |  |  |  |  |  |  |  |  |  |  |
|  | 4=Less Urgent | 0.92 | 0.90, 0.94* | 1.35 | 1.24, 1.48* | 1.56 | 1.51, 1.60* | 0.93 | 0.87, 1.00* | 1.34 | 1.12, 1.61* | 1.12 | 1.04, 1.22* | 1.37 | 1.21, 1.56* |
|  | 5=Non-Urgent | 1.00 | 0.94, 1.06 | 0.69 | 0.51, 0.93* | 2.44 | 2.28, 2.61* | 1.13 | 0.94, 1.36 | 1.82 | 1.17, 2.82* | 0.91 | 0.71, 1.16 | 1.72 | 1.24, 2.40* |
| Diagnostic Category | |  |  |  |  |  |  |  |  |  |  |  |  |  |  |
|  | Alcohol | 0.99 | 0.97, 1.01 | 0.80 | 0.73, 0.87* | 1.02 | 0.99, 1.04 | 0.94 | 0.90, 0.98* | 1.02 | 0.89, 1.17 | 1.28 | 1.23, 1.34* | 1.19 | 1.10, 1.28* |
|  | Methamphetamines | 0.89 | 0.87, 0.92* | 0.82 | 0.74, 0.91* | 0.79 | 0.77, 0.82* | 0.89 | 0.84, 0.93* | 1.08 | 0.93, 1.26 | 0.71 | 0.67, 0.76* | 1.04 | 0.96, 1.13 |
|  | Opioids | 1.01 | 0.99, 1.04 | 0.96 | 0.86, 1.07 | 1.30 | 1.26, 1.34* | 0.96 | 0.91, 1.01 | 1.93 | 1.66, 2.24* | 1.25 | 1.18, 1.33* | 1.42 | 1.28, 1.56* |
| Crowding Level | |  |  |  |  |  |  |  |  |  |  |  |  |  |  |
|  | Not crowded | Ref |  |  |  |  |  |  |  |  |  |  |  |  |  |
|  | Crowded | 0.35 | 0.34, 0.35* | 0.96 | 0.84, 1.09 | 0.89 | 0.87, 0.91* | 1.00 | 0.96, 1.04 | 1.24 | 1.07, 1.45* | 0.79 | 0.75, 0.83* | 0.52 | 0.48, 0.56* |
